# On Expectorated Matter

**Published:** 1810-03

**Authors:** George Pearson


### On Expectorated Matter. By George Pearson, M. D. F. R. S.

(Abridged from the Philosophical Transactions for 1809.)

"THE attention of physiologists has been very much withdrawn, for the last half century, from the consideration of the different states of the circulating and secreted fluids, in consequence of the opinion that the nervous and fibrous, or muscular systems, can afford satisfactory interpretations of the phænomena of living beings; and on account count of the disgust produced by the visionary properties and groundless hypotheses, originating in the humoural doctrines of Galen. But late experiments have manifested, that various things taken into the stomach can be made at pleasure to produce considerable effects, by impregnating sensibly the blood and urine, as well as the milk, sweat, and perhaps saliva. Further, the fine experiments of Professor Colman have shown, that the contagious glanders may be excited in the ass, by the transfusion of the blood of a glandered horse, and the matter from the nose of the glandered ass can produce this disease in the horse or the ass.* Hence I apprehend it is reasonable to expect, that the further investigation of the properties of the animal fluids will afford gratifying instruction to the researcher in natural science, and important practical information to the physician.

"On the present occasion, I desire the honour of communicating the knowledge I may have acquired, by investigating the properties of expectorated matter secreted by the bronchial membrane. The appearances of this substance serve to regulate the judgement of the physician concerning several diseases of the lungs; but especially of that of pulmonary tubercles, which yearly destroys 120,000 to 140,000 subjects of the United Kingdom. It is fit that I remark, that I do not notice in this paper the ingenious experiments of several learned chemists; because by so doing I should be led into a detail of too great extent for my design.

"The numerous varieties of expectorated matter, according to my observation, may be arranged and characterized under the following seven heads:

"I. The jelly-like semi-transparent kind of a blueish hue, excreted in the healthy state.

"II. The thin mucilage-like transparent matter, so copiously expectorated in broncial catarrhs.

"III. The thick opaque straw-coloured, or white and very tenacious matter, coughed up in a great variety of bronchial and pulmonary affections; especially in that of tubercles.

"IV. Puriform matter secreted without any division of continuity, or breach of surface of the bronchial mem-

* Mr. Colman alleges, that there is not a sufficient quantity of blood in a single glandered ass, to excite the glanders by the transfusion of blood into the horse.

brane, very commonly occurring in pulmonary consumptions.

" V. The matter which consists of opaque viscid masses, together with transparent fluid; or the second sort above stated, with nodules of the third or fourth kind.

" VI. Pus from the vomicæ of tubercles.

" VII. Pus from vomicæ by simple inflammation of the lungs, and without tubercles.

" Other kinds of matter are occasionally coughed up, such as calculi—masses of self coagulated lymph—serous fluid—blood itself—and perhaps the vascular substance of the lungs; but I do not write on these matters, because they either do not belong to any particular recognized disease; or they are rare occurrences in some well known disease, and are too obvious to require description.

" § I. *Sensible or obvious Properties.*

" 1. *The jelly-like matter,* as already said, is excreted in the best health, as well as sometimes in disease. It is mostly coughed, or hawked up in a morning soon after a night's repose, during which it seems to accumulate. A few masses, or nodules, then appear of the consistence of jelly, and of the size of a pea to a hazle nut. It is also at any time liable to be excreted, in consequence of various extraneous matters irritating the fauces, to the amount of a few nodules .It is of a greyish colour, or inclining to blue, with black specks; and it is rarely whitish in nodules. The consistence is that of jelly, but of much greater tenacity. It has a barely perceivable taste of common salt, or muriate of soda. It commonly floats on water, but by agitation to disengage air bubbles, it sinks. It has no smell. To the naked eye, or assisted by a single magnifier, this matter seldom appears uniform, but consists of a mixture of opaque and transparent masses of irregular figures. With the compound microscope, spherical particles were perceived, though few in number, when duly diluted. The presence of an alkali I could in no instance perceive, by means of the usual tests, namely, turmeric paper, litmus paper slightly reddened by vinegar, and cloth stained with violet juice; nor was an acid denoted by means of litmus paper, except when I had reason to believe it was derived from various acid substances taken with the food, or drink, adhering to the inside of the mouth and fauces.

" 2. *The mucilage-like expectorated matter,* according to my observation, occurs much less frequently than the other other sorts. It appears suddenly in great abundance in certain bronchial catarrhs. I have seen it to the amount of two or three pints in twenty-four hours. It is also secreted, but less copiously, in paroxysms of spasmodic asthma, and of the hooping-cough; and but rarely in pneumonic or pleuritic inflammations, and in some chronical organic diseases of the heart and lungs.

"This matter is a transparent uniform fluid of the consistence of white of egg; or of a mucilage compounded of about one part of arabic gum, and four or five parts of water. It is colourless—has a fleshy smell—has a brackish taste. After standing eight or ten hours, a deposit takes place of fibrous, leaf-like, or curdy masses, some of which are seen suspended in the clear fluid. In some cases nodules of opaque thick ropy matter, at certain times, accompany this mucilage-like matter. Under the simple magnifier I perceived irregular figured masses partly in motion and partly suspended. With the microscope, globules were seen; but larger considerably than those of the blood, and much less numerous. With the usual tests there were no indications of alkali nor of acid, provided the matter was unmixed with other things. It usually floated, or was suspended in water, when first expectorated; but on standing in the water it fell to the bottom, evidently owing to the disengagement of air-bubbles.

"By standing exposed to the air in warm weather, it sooner grew fœtid than pus of abscesses; without becoming opaque. Neither could I render it opaque or thicker, by exposure to a stream of oxygen gas for an hour; or by exposure of it in a jar of this gas for a month.

"3. *The opaque ropy matter* above mentioned.

"1st. It is secreted most copiously in that very common, and extensively epidemial disease of our climate, the *winter-cough*, occasioned by tubercles, to the amount of half a pint to a pint in twenty-four hours; especially during the winter season for several successive years, and sometimes during the whole of a long life, after the age of forty or fifty years. 2dly. It is often the expectorated matter of the pulmonary consumption of young persons, also occasioned by tubercles, but frequently mistaken for the pus of abscesses or vomicæ. 3dly. It appears, oftentimes, in pneumonic or bronchial inflammation with fever, seemingly being a beneficial discharge; as well as in some instances at the close of a fever without concomitant inflammation of the lungs. 4thly. A severe paroxysm of spasmodic asthma is often terminated in the excretion of this this kind of matter. 5thly. A secreted substance of this sort is sometimes expectorated in various chronical organic diseases of the lungs, the heart, aorta, and parts contiguous to the lungs, which occasions difficult transmission of blood through them.

"In all these instances the matter by expectoration is of the consistence of thick cream, or of thin toasted cheese; so tough as to hang in the form of a rope, four or five inches in length, on pouring it from one vessel into another. Its aggregation is such that it is readily detached in large masses from the vitreous surface of vessels. It is not unusual for small black, or reddish spots, and streaks, to appear on the surface of this sort of expectorated substance. A pretty large bulk of it is seldom throughout uniform; but it is frothy, and exhibits opaque masses of various hues with transparent matter interposed. The colour is yellowish, straw-coloured, and white, or grey; it also, though seldom, is greenish and blueish. The taste asserted by patients, is, in their own terms, various, namely, saltish, nasty, faintish, sweetish, luscious, or like that of a sweet oyster,—a sharp or sour taste is the most rare. The only smell which I have perceived is that of flesh, but very frequently there is none. When any offensive or pungent smell was perceived, immediately after expectoration, I have always found that it was owing either to the foulness of the vessel in which it was received, or it was from extraneous matters in the mouth, and from decayed teeth.

"This opaque viscid substance, being duly diluted with distilled water, was examined with microscopes of common as well as of very great powers: by means of any of them, crowds of spherical particles were seen passing to and fro in currents, not unlike those of the blood; except that they were larger. These globules I could not destroy, nor alter in form, by trituration; nor by long boiling in water; nor by exsiccation, and again dissolving in water; nor even by coagulation with mineral and vegetable acids, with alcohol, with sulphuric ether, or with tannin, and alum; nor by mixture with caustic alkalies in a proportion which leaves the liquor turbid; nor for some time after the putrefactive process had appeared. But these globules disappear with such a proportion of sulphuric acid as detaches charcoal; or of nitric acid, and of liquid potash, as produce a clear solution; also by charring by fire. It is perhaps superfluous to remark, that these atomic globules are quite different from the air bubbles usually entangled in in this kind of matter, as perceived by the microscope; the latter differ much from the former, in being of far greater magnitude—in being less numerous—in being transparent, and on disappearing on agitation, or heating the matter, or even by mere standing.

" For the most part this expectorated substance swims on water; but by agitation or stirring to disengage air bubbles, or by merely standing, it sinks. Some of the lumps suddenly hawked up, immediately fall to the bottom of a vessel of water. No signs of either acid or alkali, appeared on the trials of this matter with well known reagents, provided it was free from extraneous matter; but it was apt to betray acidity from things taken with the food or drink.

" 4. *Puriform matter.* I have seen this matter expectorated in several diseases in the quantity of two or three ounces to half a pint in twenty-four hours, on some rare occasions, without any breach of surface. I believe it would be considered by every one to be *pus*, having the properties commonly admitted to be those of this substance. It will, however, perhaps, only be just to call it *puriform*, for the present, as it appears to me probable, that I shall hereafter be able to show that it possesses properties not belonging to pus of abscesses, although in the obvious, or sensible properties, it is similar to such pus. Accordingly this expectorated matter is not only opaque, white, or yellowish, and thick as the richest cream, but it also has not more tenacity than cream. It is not apt to entangle air, and therefore it immediately mingles with water, rendering it milky; and presently subsides to the bottom, leaving the water clear, or at least whey-coloured. It appears to the naked eye uniform in its texture; and nearly so under the simple lens: but under the microscope thousands of globules similar to those of the blood are seen, which are indestructible as those above related belonging to another kind of expectorated matter.

" The substance, of which I am now speaking, is most frequently excreted in the latter stages of pulmonary phthisis, for many weeks successively. It is taken for granted that this matter is from a breach of surface or ulceration; but on examination after death, such a state was not found, in many instances, under my observation, although the lungs were as usual full of tubercles and vomicæ. This puriform matter is occasionally expectorated in certain other diseases. The last summer my colleague, Dr. Nevinson, furnished me with several ounces of this sort of substance, substance, but of a greenish hue, and of the consistence of thin cream; which was expectorated by a woman in the third week from the attack of the measles. In a few days she died. On examination of the lungs very carefully, by the excellent house surgeon of St. George's hospital, Mr. Dawes, no ulceration could be discovered in the trachea or in the bronchial tubes; nor were any tubercles or abscesses found in the lungs. The patient, according to my information, had expectorated more than a pint of this fluid every twenty-four hours for a week before death. In another hospital case, a man laboured under a cough with spitting of matter, which all who saw it called pus, and as usual it was considered to arise from an ulceration, or suppurated tubercles; but, on examination after death, the disease was ascertained to be condensation of the lungs, to the consistence of liver, with water in the cavities of the chest, and nothing more.

"5. *Opaque viscid matter of a third, and perhaps fourth sort,* above distinguished, appearing in nodules, and irregular-figured masses, *mixed with transparent slimy matter of the second sort.*

"It is not unusual to see the mixture of these two different kinds, from severe fits of coughing in that constant epidemy of the British islands, the winter chronick pneumonia.

"Different parts of the bronchial membrane being in different states, may account for the secretion of the two different matters. This seems more probable than that these different matters should be secreted from the same part; although it is true that the same part does secrete at one period transparent thin slime, and at another an opaque thick matter. The former is occasioned by great irritation of the membrane, and the latter is the effect of a more gradual secretion with much less irritation.

"For the sake of brevity, I avoid a further description. The practical application of these observations, however important, would not be suitable in this place.

"The sixth and seventh kinds of expectorated substances being secreted after a quite different manner, and being very different in their nature from the preceding five kinds, I shall not give an account of them in this paper."

[The author then describes at large, and with much precision, the effects produced on expectorated matter by the agency of caloric; of alcohol of wine, of water; and of acetous acid—also some experiments with different objects from any of these; but we confine the remaining part of the the present extract to his *Conclusions*, as containing that kind of information which will be most acceptable to the generality of our readers.]

" *Conclusions.*

" 1. From the preceding experiments and observations, and from others which I might have related, it does not appear that the various kinds of expectorated matter, page 215, differ in the ingredients of their composition, but merely in the proportion of them to one another.

" 2. It has been shewn that expectorated matter consists of coagulable, or, as it is also now frequently termed, *albuminous* animal substance, and of water impregnated with several saline and earthy bodies;—that the largest proportion of the animal substance, which may justly be called an oxide, amounts to one twelfth, and in some very rare cases to one-tenth of the expectorated matter, reduced to a brittle state by evaporation; and that the smallest proportion of this oxide, in rare instances, amounts to one forty-fifth of the expectorated matter; but that the usual proportions of it vary between one-twentieth and one-sixteenth of this coagulable oxide to the evaporable water, that is, between five and six per cent. of the expectorated matter.

" 3. The impregnating substances have been shown to be muriate of soda, varying commonly between one and a half to two and a half per 1000 of the expectorated matter—Potash varying between one-half and three-fourths of a part per 1000—Phosphate of lime about half a part of 1000—Ammonia, united probably to the phosphoric acid; phosphate, perhaps of magnesia; carbonate of lime; a sulphate; vitrifiable matter, or perhaps silica; and oxide of iron. But the whole of these last six substances scarcely amounting to one part in 1000 of the expectorated matter, it would be useless to estimate the proportion of each of them. It is very probable that the proportions and quantities of these ingredients vary much more than now represented in different states of disease and health.* It is very probable also, that some of the ingredients may occasionally be absent, and others of a different kind be present, agreeably to

* In one case, the opaque expectorated matter in a pulmonary consumption having been exsiccated to brittleness, became almost liquid after a night's exposure to the air.

to the different states, on different occasions, of the other secretions.

"4. It is manifest that the different states of consistence of expectorated matter are owing to the proportion of albuminous or coagulable oxide; but I purposely avoid giving an account of the different conditions of health, on which the differences of consistence depend.

"5. The thicker the matter, the smaller I commonly found the quantity of saline impregnation. Hence, in sudden and copious secretions of the bronchial membrane, the matter is asserted to be salt, and to feel hot. In such instances, the proportion of coagulable matter was small, but that of the saline impregnations, particularly of the muriate of soda, and neutralized potash, so great, that the exsiccated expectorated substance tasted very salt, and presently grew moist, or even partially deliquesced; but the opaque ropy or puriform matter afforded a much larger proportion of exsiccated residue, which was but slightly salt, and generally only became soft on exposure to the air. This property of growing moist depends upon the potash.

"6. Each of the human fluids, according to my experiments, contains neutralized potash; at least, this is the fact of the blood, dropsy fluid, pus of abcesses, and pus secreted without breach of surface; the fluid effused by vesicating with cantharides; the urine; and in course in the very abundant secretion from the nose by a catarrh. The alkali being united to oxide of animal matter in these fluids, it is easily demonstrable.

"7. Although I think I have discovered many properties by which expectorated secretion may be distinguished from expectorated pus, I shall not speak of them, on this occasion, further than just to observe that the saline impregnation of pus, particularly that of potash, and muriate of soda, is in very much less proportion than in expectorated secretion; and hence it does not become moist after exsiccation, on exposure to the air.

"8. It has been, I believe, uniformly asserted, that the circulating and secreted fluids are impregnated with soda; that it is especially in the matter secreted by the bronchial membrane. The experiments of others must confirm or disprove mine. It seems, however, much more reasonable, that the human fluids should be found to contain potash than soda, united to some oxide or destructible acid; because the former alkali is daily introduced with the vegetable food, and with the drink of fermented liquor; and it is is as little likely to be destroyed, as the muriate of soda also induced in the very same way. But our food and drink do not, commonly at least, contain the soda united to a destructible acid, or an oxide.

"9. It is plain, from the preceding experiments, that expectorated matter belongs to the class of coagulable fluids and not of gelatinizable, or, as commonly asserted, mucous fluids. It differs from the coagulable fluid, serum of blood, in forming a much thicker fluid with a much larger proportion of water: for serum, and also the water of blisters, is quite liquid, although they afford, on exsiccation, one-twelfth to one-eleventh of their weight of brittle residue, while some kinds of expectorated matter, of the consistence of mucilage, afford only one-fortieth of dry residue, and others of the consistence of thin paste afford only one-fourteenth of residue.

"10. But for the unavoidable extent of this paper, I should trouble the learned Society with various other conclusions and remarks, especially concerning the *globularity* of expectorated matter, which seems to indicate organization. Although Antonius Van Lewenhoeck, above a century ago, discovered the globularity of the blood, and even noticed it in other animal fluids, neither he, nor any other person, as far as I know, investigated the subject in any fluid but the blood, till, by Mr. Home's acuteness and industry, at a very early period of life, it was observed in pus. I have in this paper related, that expectorated matter, especially the opaque ropy kind, as well as the puriform, is full of globules; and that, except by such agents as destroy charcoal, they are scarcely destructible. Do these spherical particles consist chiefly of organized carbonaceous matter?"

